# Genome-Wide Analysis of Smad7-Mediated Transcription in Mouse Embryonic Stem Cells

**DOI:** 10.3390/ijms222413598

**Published:** 2021-12-18

**Authors:** Guohua Meng, Andrea Lauria, Mara Maldotti, Francesca Anselmi, Isabelle Laurence Polignano, Stefania Rapelli, Daniela Donna, Salvatore Oliviero

**Affiliations:** 1 Dipartimento di Scienze della Vita e Biologia dei Sistemi and MBC, Università di Torino, Via Nizza 52, 10126 Turin, Italy; guohua.meng@unito.it (G.M.); andrea.lauria@unito.it (A.L.); mara.maldotti@unito.it (M.M.); francesca.anselmi@unito.it (F.A.); isabelle.laurence.polignano@unito.it (I.L.P.); stefania.rapelli@unito.it (S.R.); daniela.donna@unito.it (D.D.); 2 Italian Institute for Genomic Medicine (IIGM), Sp142 Km 3.95, Candiolo, 10060 Turin, Italy

**Keywords:** embryonic stem cells, Smad7, transcription

## Abstract

Smad7 has been identified as a negative regulator of the transforming growth factor TGF-β pathway by direct interaction with the TGF-β type I receptor (TβR-I). Although Smad7 has also been shown to play TGF-β unrelated functions in the cytoplasm and in the nucleus, a comprehensive analysis of its nuclear function has not yet been performed. Here, we show that in ESCs Smad7 is mainly nuclear and acts as a general transcription factor regulating several genes unrelated to the TGF-β pathway. Loss of Smad7 results in the downregulation of several key stemness master regulators, including *Pou5f1* and *Zfp42*, and in the upregulation of developmental genes, with consequent loss of the stem phenotype. Integrative analysis of genome-wide mapping data for Smad7 and ESC self-renewal and pluripotency transcriptional regulators revealed that Smad7 co-occupies promoters of highly expressed key stemness regulators genes, by binding to a specific consensus response element NCGGAAMM. Altogether, our data establishes Smad7 as a new, integral component of the regulatory circuitry that controls ESC identity.

## 1. Introduction

Pluripotency is the ability to produce all cell types and is the defining characteristic of embryonic stem cells (ESCs) (cultured cells derived from the inner cell mass of mammalian blastocyst) [1,2]. ESCs self-renew infinitely in vitro while maintaining their pluripotency. The pluripotency transcription factors play critical roles in maintaining ESC identity by forming complex regulatory circuits, in which they cooperatively maintain the pluripotency by self-activating and silencing lineage-specific regulators [3,4]. Transforming Growth Factor beta (TGF-β) pathway activation in mouse embryonic stem cells (ESCs) shows opposed roles in maintaining pluripotency and differentiation [5]. Members of the TGF-β superfamily, including TGF-β, Activin A, Nodal, and bone morphogenetic proteins (BMP), play a significant role in maintaining pluripotency in stem cells and controlling lineage-specific differentiation [6,7]. The activation of the TGF-β branch leads to the recruitment of receptor-regulated Smad2 and Smad3 (R-SMAD), which form complexes with Smad4 (Co-SMAD) and move into the nucleus to regulate target gene transcription [8,9,10]. Smad7, a major inhibitory SMAD protein (I-SMAD), is induced by all the ligands of the TGF-β superfamily and acts as negative feedback of TGF-β signaling by interacting with type I receptors and inhibiting the phosphorylation of targeting receptors [11,12,13,14,15,16,17].

However, it has also been reported that in ESCs *Smad7* is upregulated by the transducer and activator of transcription 3 (STAT3) and NANOG and in turn it activates gp130-mediated STAT3 signaling to maintain ESCs in the undifferentiated state [18,19] suggesting that, besides the feedback loop of the TGF-β signaling, Smad7 could play other functions. In line with this hypothesis, it has been shown that, Smad7 promotes mouse hematopoietic stem cells self-renewal [20]. In Hep3B cells Smad7 interferes with R-Smads/Smad4-DNA association [21]. In prostate cancer cells, Smad7 has been shown to bind to the *c-Jun* and *HDAC6* regulatory regions in the absence of Smad factors to upregulate these genes [22].

To verify the hypothesis that Smad7 plays a general function in the nucleus, we analysed the gene expression profile of Smad7-regulated genes and performed a genome-wide binding analysis of Smad7 in mouse ESCs by ChIP-seq. Integrative analyses of chromosomal targets on the ESC genome shows that Smad7 binds DNA on promoters co-occupied by either the E2F1, Myc, and/or Smads. Altogether, we propose that Smad7 is a *bona fide* transcription factor involved in ESC self-renewal and pluripotency maintenance.

## 2. Results

### 2.1. Nuclear Smad7 Regulates Genes Involved in ESC Self-Renewal and Pluripotency Maintenance

**Figure 1:**
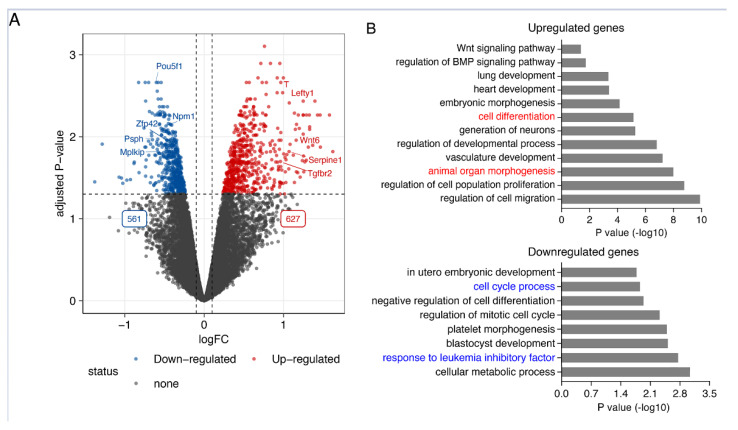
Smad7 regulates the expression of the genes involved in pluripotency maintenance and self-renewal in ESCs. (**A**) Volcano plot showing the 1188 differentially expressed genes. Significantly differentially expressed genes in response to Smad7 KD (FDR < 0.05 and log2 fold change > 0.2) are indicated in red and blue. While 627 genes are upregulated, 561 genes are downregulated. The dashed line indicates the *p*-value significance threshold. Four biological replicates were used for this analysis. (**B**) Bar graph representing the enriched GO terms of biological processes in upregulated and downregulated genes after the KD of Smad7.

Smad7 has been previously demonstrated to promote mouse ESC self-renewal and pluripotency maintenance binding to the leukemia inhibitory factor (LIF) coreceptor glycoprotein 130 (gp130) [19]. To identify Smad7-regulated genes, we first performed Smad7 knockdown (KD) using 2 independent short hairpin RNAs (shRNAs). We confirmed Smad7 knockdown by RT-qPCR, western blot and immunofluorescence assay (Figure S1A–D). Smad7 downregulation resulted in a switch from an epithelial-like cell morphology to mesenchymal-like and decreased alkaline phosphatase (AP) activity (Figure S1E,F). RNA-seq of four biological replicates of Smad7 KD (Figure S1G,H) identified 1188 differentially expressed genes of which 627 genes were significantly upregulated, and 561 downregulated (Figure 1A and Figure S1).

Smad7 is a well-established negative regulator in TGF-β/SMAD signaling. In line with this function, among the upregulated genes, other genes were involved in this signaling pathway (i.e., *Bmp1*, *Lefty1*, *Serpine1*, *Tgfbr2*, and *Tgfbr3*). In agreement with the observed cell phenotype, gene ontology analysis of the upregulated genes revealed an enrichment for genes related to cell and organ differentiation (Figure 1B top panel), including genes involved in neural development (i.e., *Celsr1*, *Fgf8*, *Id2*, *Smarca2*, *Wnt6*, *Wnt9a*, and *Wnt11*), meso-endoderm differentiation (i.e., *Cxcr4*, *Gata6*, *Kdr*, *T*, and *Wls*), and epithelial-mesenchymal transition (EMT) (i.e., *Nfatc1*, *Serpine2*, *Lrp6*, *Col4a1*, *Eng*, *Ecm1*, and *Notch1*).

Concordantly, downregulated genes were enriched in the cellular metabolic process, response to LIF, and cell cycle progression (Figure 1B bottom panel), including well-known genes involved in ESC self-renewal and pluripotency maintenance (i.e., *Pou5f1*, *Ccnd1*, *Psph*, and *Ptma*). These results indicate that Smad7, besides its established function of inhibiting TGF-β/SMAD signaling, plays a key role in controlling the expression of the genes involved in ESC identity.

### 2.2. Genome-Wide Identification and Characterization of Smad7 Binding Sites in ESCs

**Figure 2:**
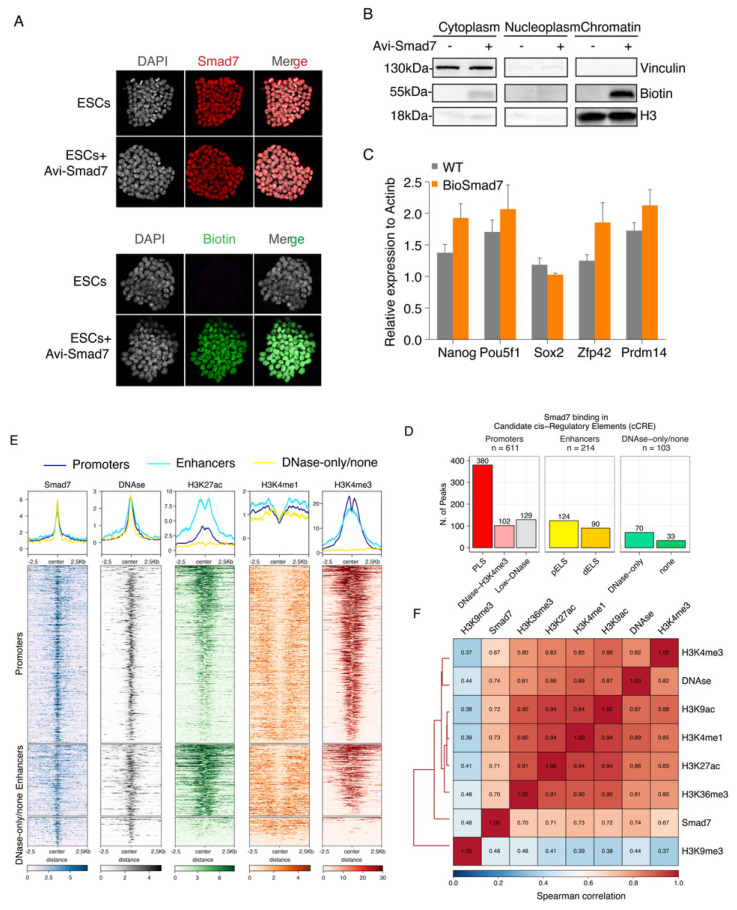
Genome-wide identification and characterization of Smad7 binding sites in ESCs. (**A**) Immunofluorescence assay of Smad7 (up) and Biotin (down) in the ESCs transfected with Avi-Smad7 compared with the un-transfected control. Nuclei were stained with DAPI. (**B**) Western blot analysis using an anti-Biotin antibody compared the expression of exogenous (bio-tagged) Smad7 in three cells’ fractions. BirA cells and Avi-Smad7 transfected cells were used in this experiment. Vinculin was used as a cytoplasm marker, and H3 was used as a chromatin marker. (**C**) RT-qPCR analysis of *Nanog*, *Pou5f1*, *Sox2*, *Zfp42*, and *Prdm14* in Avi-Smad7 transfected cells (BioSmad7) in comparison to WT ESCs. The expression level was normalized to *Actinb*. Data are shown as mean ± SEM; *n* = 3. (**D**) A bar graph presenting the distribution of Smad7 binding sites in candidate *cis*-regulatory elements (cCRE). cCREs with PLS fall within 200 bp (center to center) of an annotated GENCODE TSS and have high DNase and H3K4me3 signals; The subset of cCREs-ELS within 2 kb of a TSS is denoted proximal (cCRE-pELS), while the remaining subset is denoted distal (cCRE-dELS); DNase-H3K4me3 cCREs have high H3K4me3 max-Z scores but low H3K27ac max-Z scores and do not fall within 200 bp of a TSS; DNase-only group contains cCREs with high DNase Z-scores but low H3K4me3 and H3K27ac. PLS: promoter-like signatures; pELS: proximal enhancer-like signatures; dELS = distal enhancer-like signatures. (**E**) Heatmaps show the colocalization of Smad7 and DNAse, H3K27ac, H3K4me1, H3K4me3. Occupancy signals within ± 2.5 Kb of the center of Smad7-binding sites are shown. Note the presence of Smad7 both at active enhancers (H3K4me1 positive) and active promoters (H3K4me3 positive). Aggregation plots illustrating the fraction of relative midpoint positions of the ChIP-seq peaks of Smad7 are shown above the heatmaps. (**F**) Hierarchical clustering of pairwise Spearman correlation of Smad7 and ChIP-seq datasets indicated. Colors indicate the level of correlation (red indicates perfect correlation, blue indicates ideal anticorrelation). Smad7 clusters together with active histone marks.

To date, no study has investigated the role of Smad7 as a transcriptional regulator. In different cell lines, Smad7 is predominantly localized in the nucleus and translocated to the cytoplasm only upon TGF-β receptor activation [21,23], suggesting that Smad7 may play a regulatory role in the nucleus. To determine whether Smad7 is involved in stem cell maintenance by direct transcriptional regulation, we sought to analyse, for the first time, Smad7 binding to the genome via ChIP-seq. To this end, we generated an Avitag-tagged *Smad7* by labelling the N-terminus of *Smad7* with the Avitag; upon co-expression of the construct with the BirA protein for *E. coli*, Smad7 is biotinylated *in vivo*, enabling ChIP-seq via streptavidin capture, under very stringent conditions (Figure S2A–E) [3,4]. Avi-Smad7 expression assessed by Smad7 and Biotin immunofluorescence analyses showed expression levels comparable to endogenous nuclear Smad7 (Figure 2A). As shown in Figure 2B, the biotinylated Smad7 was highly enriched in the chromatin fraction. RT-qPCR analysis of core pluripotency factors (*Nanog*, *Pou5f1*, and *Sox2*) and ESC naïve state markers (*Zfp42* and *Prdm14*) showed that cells expressing Bio-Smad7 were functionally equivalent to wild-type ESC stem-cell marker genes expression (Figure 2C).

We performed ChIP-seq experiments in two independent biological replicates (Figure S3A–E). Principal component analysis (PCA) and correlation analyses both showed good agreement between the two replicates (Figure S3F). Smad7 mapping on the genome identified a total of 928 high-confidence peaks in both replicates (Table S1; Figure S2). Interestingly, the most significant enrichment (*p*-value < 1 × 10^−16^) was observed for peaks residing within promoters, accounting for approximately half of the Smad7 peaks (48%), and enhancers (23.1%) (Figure S4B–D). 

Approximately 40% of Smad7 binding peaks were localized in regions marked by H3K4me3, DNAseI hypersensitivity sites (DHSs) within 200 bp of annotated transcriptional start sites (TSSs) (Figure 2D,E and Figure S4A,D). Accordingly, clustering analysis showed a strong colocalization of Smad7 with the H3K4me3 mark (Figure 2E,F). Association of Smad7 binding to *cis*-regulatory regions showed that about 23% of target sites were with enhancer-like signatures (ELS), including 13.4% proximal enhancer-like signatures (pELS) and 9.7% distal enhancer-like signatures (dELS) of known TSSs, in agreement with the clustering analysis showing a strong colocalization of Smad7 with the H3K27ac mark (Figure 2D–F and Figure S4D,F).

### 2.3. Smad7 Binds to Promoters of Genes Involved in ESC Self-Renewal

**Figure 3:**
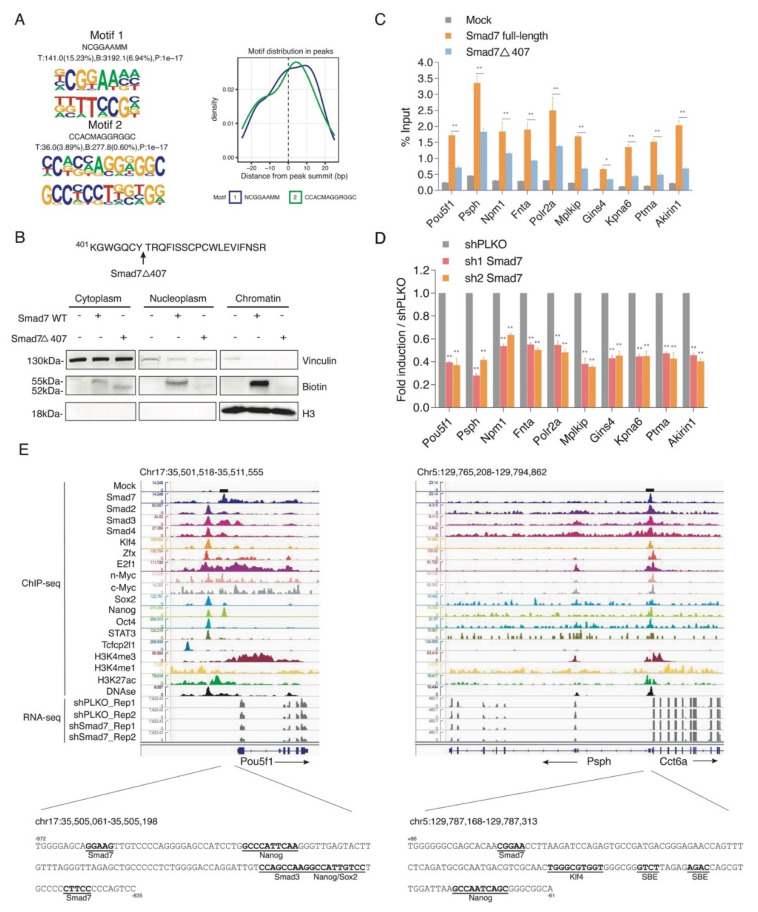
Smad7 binds to the promoters of genes involved in ESC self-renewal. (**A**) Left panel: *De novo* DNA sequence motifs and their STAMP logos and enrichment statistics, including observed binding sites. Occupancy signals within ±25 bp of the Smad7 peak summit are shown. (**B**) Top panel: Schematic of the mutant of Smad7 (Smad7Δ407), which lacks the last 19 amino acids at the C terminal. Smad7Δ407 construct also has an Avi-tag at its N terminal. Bottom panel: Western blot showing the subcellular fractionation of Smad7 WT and Smad7Δ407. Biotin antibody was used to compare the expression of exogenous (bio-tagged) Smad7 WT and Smad7Δ407 in three fractions of cells. BirA cells, Smad7 WT, and Smad7Δ407 transfected cells were used in this experiment. Vinculin was used as a cytoplasm marker, and H3 was used as a chromatin marker. (**C**) RT-qPCR analysis of BioSmad7 WT and Smad7Δ407 ChIP samples at the promoters of the indicated genes. The results are shown as the percentage (1/100) of the input. Data are shown as mean ± SEM; *n* = 3. ** *p* < 0.01. * *p* < 0.05. (**D**) RT-qPCR analysis of the indicated transcripts upon a knockdown (KD) of Smad7 proteins. The results are shown as fold difference normalized to shPLKO. Data are shown as mean ± SEM; *n* = 3. ** *p* < 0.01. (**E**) Representative genomic occupancy profiles of genes identified by BioSmad7 ChIP-Seq. IGV images showing the comparison of ChIP-seq peaks of Smad7 binding (current study), other 14 factors (accession code GSE11431, GSE125116), the locations of histone H3 modifications (accession code GSE12241, GSE11172, and GSE31039), DNAse, and RNA-seq of two independent experiments of Smad7 knockdown (current study) at genes downregulated by Smad7 knockdown. Two examples (*Pou5f1* and *Psph*) are shown. The genes and their direction of transcription are indicated by arrows. The binding loci of Smad7 are indicated by black boxes. Putative transcription factor binding sites are annotated in bold black.

Motif discovery analysis on Smad7 ChIP-seq dataset showed that Smad7 binding sites were enriched for the consensus NCGGAAMM (Figure 3A).

Next, the integrative analysis of RNA-seq and ChIP-seq data showed approximately 6% Smad7-promoter bound genes were down-modulated by Smad7 knockdown (Figure S5A,B). Gene ontology analysis showed enrichment in cellular response to LIF, chromatin remodelling, gastrulation, and embryo development (Figure S5C).

A selection of ChIP-seq Smad7 target genes involved in pluripotency and self-renewal activity, including *Pou5f1*, activated phosphoserine phosphatase *Psph* [24], the cell proliferation regulator *Akirin1* [25], cell cycle regulator *Mplkip* [26], and cell apoptosis inhibitor *Ptma* [27], and target genes involved in the cell cycle and cell metabolism process, including the general ESC transcription factor *Polr2a* [28], serine-threonine kinase receptor *Fnta* [29], and the transcription co-activator *Npm1* [30], were verified by site-directed ChIP in ESCs transfected with Avi-Smad7 WT or with a mutant deletion of the last 19 amino acids at its C-terminus (Avi-Smad7Δ407), previously described to localise in the cytoplasm fraction [23] (Figure 3B). ChIP analysis showed a significant reduction in the Smad7 mutant binding with respect to the WT protein (Figure 3C). Analysis of the transcripts performed by RT-qPCR in Smad7 silenced ESCs showed that Smad7 binding reduction corresponds to a downregulation of the Smad7 bound genes (Figure 3D), thus indicating that Smad7 binding contributes to the transcriptional activation of its target genes. As examples, the binding profiles for Smad7 compared with different ESC factors, the active histone marks, and the RNA-seq of Smad7 KD at the *Pou5f1* and *Psph* gene loci are shown in Figure 3E. Analysis of the Smad7 binding at a several target sites identified by ChIP-seq confirmed the specific Smad7 recruitment to the genome.

### 2.4. Smad7 Participates to ESC Regulatory Network

**Figure 4:**
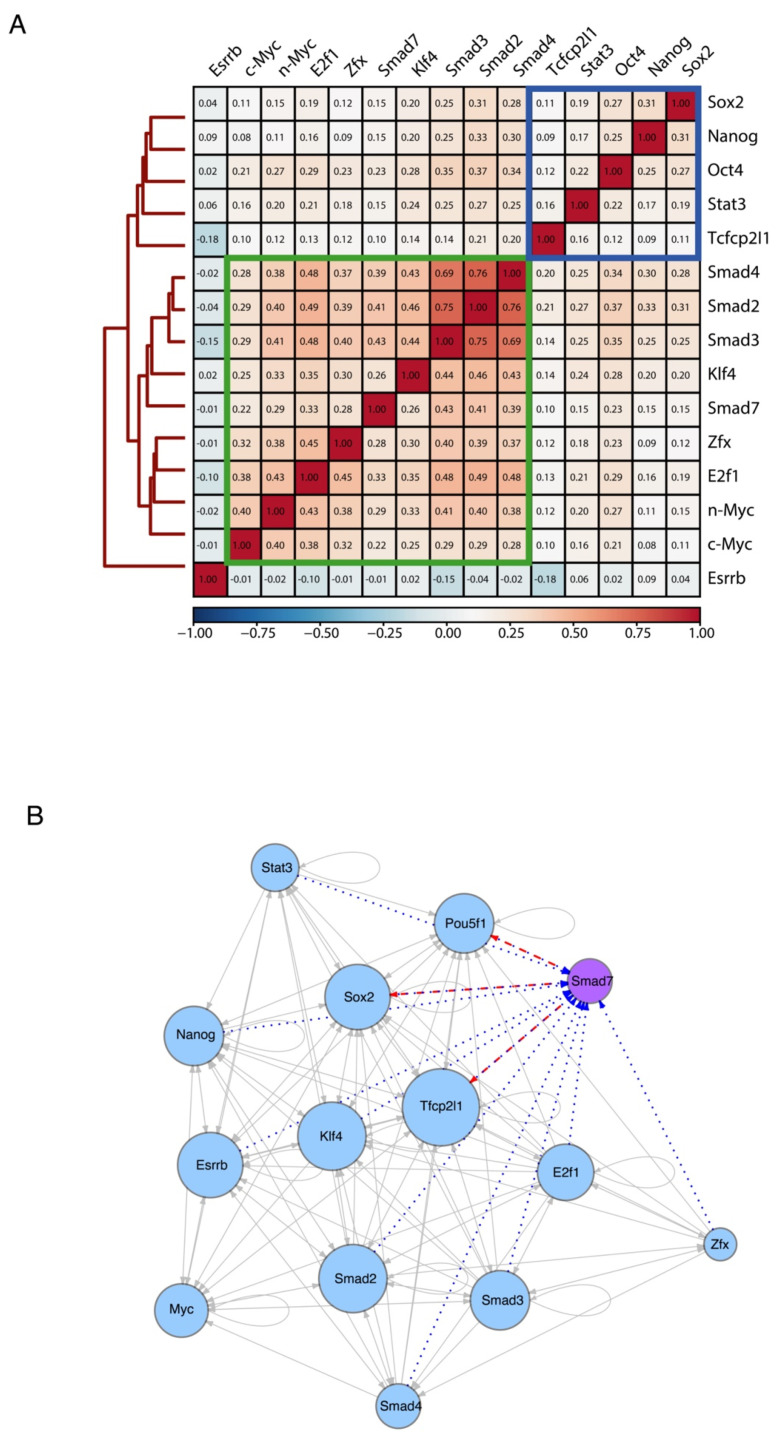
Smad7 participates in ES-Cell Regulatory Network. (**A**) Heatmap showing the similarity of their binding sites among diverse transcription factors and coregulators (accession code GSE11431, GSE125116) as well as Smad7 (current study) in mouse ESCs. Colors in the heatmap show the level of overlap for each pair of samples (red, all binding sites overlapped; orange, overlap expected by chance; blue, mutually exclusive binding). Core transcription factors are boxed in blue. Myc cluster is boxed in green. (**B**) Transcriptional network of regulatory interactions predicted from ChIP-seq binding assays. Nodes are ChIP-seq-assayed transcription factors (Smad7 (purple node) is from the current study, other factors (blue nodes) are from published datasets: accession code GSE11431, GSE125116). The size of each circle represents the degree of the interaction among different factors. Arrowhead indicates the direction of transcriptional regulation. The transcriptional regulation by Smad7 is highlighted by two different patterns: the red dash line indicates the regulation starting from Smad7 (i.e., Pou5f1, Sox2, and Tfcp2l1), and the blue dot line represents the regulation towards Smad7 (i.e., Pou5f1, Sox2, Tfcp2l1, Stat3, Nanog, Esrrb, Klf4, Smad2, Smad3, Smad4, E2f1, and Zfx).

Next, we compared the genomic target sites of Smad7 with the binding profiles of a panel of well-known ESC-specific transcription factors (TFs), using publicly available ChIP-seq experiments (Nanog, Oct4, Sox2, Stat3, Zfx, c-Myc, n-Myc, Klf4, Esrrb, Tcfcp2l1, E2f1, and Smad2/3/4) [3]. To assess the genome-wide binding preferences of Smad7 versus other regulatory factors in ESCs, we calculated target co-occupancy scores as average ChIP-seq signals on the merged set of genomic regions bound by each of the factors (see Methods). Hierarchical clustering of TFs scores revealed that, overall, Nanog, Oct4, and Sox2 core TFs co-occupy genomic target loci, whereas Smad7 binding mainly co-occurs with the TF group including c-Myc, n-Myc, Klf4, Zfx, E2f1, and Smad2/3/4 (Figure 4A and Figure S4E). Interestingly, we found known transcription factors enriched in the proximity of the Smad7 binding sites (Figure S5D).

We then inferred the regulatory interactions between the pluripotency-associated TFs by constructing a TF-target network from the binding profiles of each analyzed factor (see Methods). In Figure 4B, a directed edge points from the TF to each of its targets (including self-regulatory loops), and the number of interactions is described as the size of each node, reflecting the TFs co-occupancy degree of the promoter and/or enhancer associated to the gene encoding each factor. The resulting network model depicts the interconnection of Smad7 within the regulatory circuitry controlling ESC identity. Taken together, the above results indicate that Smad7 binds to the genome to key target genes contributing to the regulation of ESC self-renewal and pluripotency.

## 3. Discussion

Smad7, a gene recognized mainly as a negative feedback product of TGF-β superfamily signaling, has been shown to play regulatory roles distinct from its antagonistic effect on TGF-β pathway [19], and also in the nucleus in different cell types [21,22]. 

**Figure 5:**
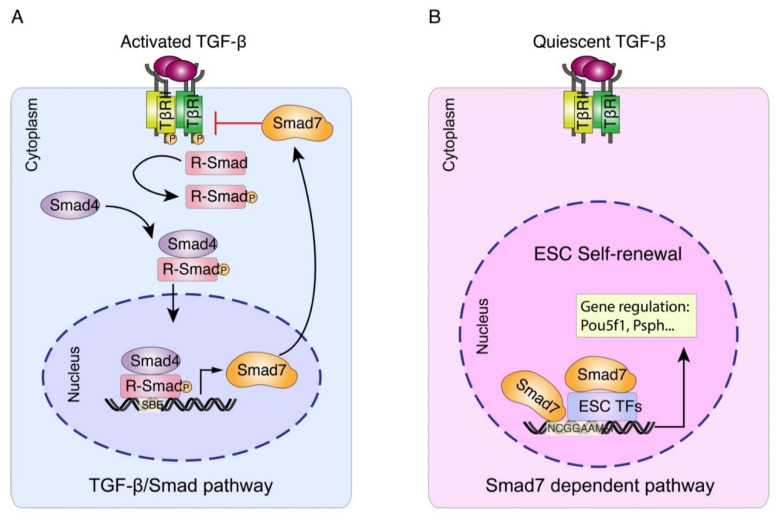
Working model for Smad7-mediated transcriptional regulation in mESC. (**A**) Upon TGFβ/Smad pathway activation, Smad7 can function as a TGFβ antagonist in the cytoplasm [11,12]. (**B**) Our study illustrates Smad7 functions as a transcription factor and regulates the target gene expression independently or by interacting with other ESC factors in the nucleus, thus promoting ESC self-renewal and pluripotency maintenance.

Here we report that, in addition to its known cytoplasmic regulatory function, Smad7 is also a *bona fide* transcription factor that in ESCs binds the DNA and activates the transcription of its target genes (Figure 5). Smad7 participates to the ESC regulatory network to contribute to ESC self-renewal and pluripotency. RNA-seq analysis in ESC Smad7 knockdown revealed that Smad7 regulates a number of genes unrelated to the TGFβ/SMAD signaling. Importantly, Smad7 silencing significantly decreased the expression of ESC self-renewal and pluripotency markers and increased the expression of several genes involved in the neurectoderm and meso-endoderm differentiation. These results are in line with studies demonstrating that Smad7 is regulated by Stat3 and Nanog [18], indicating that Smad7 is a downstream effector of these genes to contribute to the maintenance of mouse embryonic stem cells in the pluripotent and undifferentiated state in a LIF dependent manner.

Genome-wide ChIP-seq analysis revealed that in ESCs Smad7 binds preferentially to sites associated with promoter and enhancer regions marked by epigenetic modifications corresponding to actively transcribed regions, and its silencing results in the downregulation of the target genes, indicating that Smad7 is a general positive TF.

It was previously demonstrated that in ESCs many TFs cluster on functionally important regulatory loci and tend to co-occur in two distinct groups. A first group includes ESC core factors (i.e., Oct4, Nanog, Sox2, and Stat3) and a second group includes factors involved in cell-cycle progression and reprogramming (i.e., Myc, E2f1, and Klf4) [3]. We found that Smad7 is preferentially associated to the second group to bind a number of genes involved in pluripotency in regulatory regions bound by many other factors.

Interestingly, Smad7 binds to the proximal element of Pou5f1 and together with many other transcription factors binding to this regulatory region contributes to its expression in ESCs.

Other important ESC self-renewal genes including *Kpna6*, *Akirin1*, *Myb*, *Nanog*, *Sox2*, *Dnmt3b*, *Id2*, *Tbx3*, *Sall4*, *Myc*, *Zfp398*, *Klf5*, and *Ets1* were identified as Smad7 target genes (Figures S3 and S4).

Motif discovery analysis showed that Smad7 had a preferential binding consensus (NCGGAAMM), which is distinct from that of the other members of the Smad family, such as Smad2/3 preferentially bind to G(T/G)CT(G). This difference could be due to the fact that Smad2/3 bind the DNA via MH1 domain, while Smad7 has been shown to bind DNA via its MH2 domain [9,21]. It has also been observed that Smad factors are known to bind the DNA in cooperation with many other transcription factors [9]. We cannot exclude that Smad7 cooperates with other factors to bind DNA. Further analyses will clarify this point.

In sum we showed that Smad7 is a *bona fide* transcription factor highly interconnected with known pluripotency factors of the ESC regulatory network. Our results expand the ESC network and reveal that the ESC interconnectivity of the regulatory factors is larger than previously expected.

Recent evidence points out that Smad7 activity, plays a role in the control of neoplastic processes in different organs also independently from its function of TGF-β signaling repression [31,32,33,34,35]. Our results might contribute to the clarification of the Smad7 role in tumorigenesis.

## 4. Materials and Methods

### 4.1. Embryonic Stem Cell Culture

E14 mouse ESCs were grown on 0.1% gelatin-coated plates and maintained in high-glucose DMEM (Invitrogen, Waltham, MA, USA) supplemented with 18% EmbryoMax^®^ FBS (Millipore, Burlington, MA, USA), 0.1 mM non-essential amino acids (Invitrogen, Waltham, MA, USA), 1 mM sodium pyruvate (Invitrogen, Waltham, MA, USA), 0.1 mM 2-mercaptoethanol, 1000 U/mL Leukemia Inhibitory Factor (LIF; Millipore, Burlington, MA, USA), 25 U/mL penicillin, and 25 μg/mL streptomycin. Cells were routinely tested to be free of mycoplasma.

### 4.2. DNA Construct and shRNA

pEF1α-BirA-V5 construct expresses C-terminal V5-tagged BirA biotin ligase. The full-length *Smad7* cDNA and mutant (*Smad7*Δ407; it lacks 19 amino acids at its C-terminus) were obtained by PCR amplification and N-terminally tagged by Avitag. Then the expression constructs were cloned into the Not I and Xba I sites of the pEF6 vector (Invitrogen, Waltham, MA, USA).

Smad7 shRNAs were constructed using the TRC hairpin design tool (http://www.broadinstitute.org/rnai/public/seq/search (accessed on 17 December 2021)) targeting the following sequences:

5′-GCTTTCAGATTCCCAACTTCT-3′ (shRNA1 Smad7);

5′-GTCTTGTTCTTTGAGAAATTA-3′ (shRNA2 Smad7)

Annealed oligonucleotides were cloned into pLKO.1 lentiviral vector (Addgene:10879), and each construct was verified by sequencing.

### 4.3. Transient Transfection

Transient transfection was performed using Lipofectamine 2000 Transfection Reagent (Invitrogen, Waltham, MA, USA) for mouse ESCs in accordance to the recommendations of the manufacturer. For *Smad7* knockdown, cells were transfected twice with 5 μg of the specific shRNA construct, and maintained in growth medium for 48 h.

### 4.4. In Vivo Biotinylation

ESCs stably expressing *Escherichia coli biotin* ligase BirA enzyme was generated according to previous publication [36]. In brief, E14 ESCs were transfected with a plasmid construct expressing BirA-V5 and cultured for ten days in a growth medium with G418. Clones were picked and expanded for western blotting by using anti-V5-HRP (Invitrogen, Waltham, MA, USA). A BirA-expressing ES clone was then used for transfection with plasmid construct containing biotin-tagged *Smad7* cDNA. ESCs expressing tagged Smad7 were identified by western blotting with anti-Biotin of the total lysates.

### 4.5. Subcellular Fractionation

Cytoplasmic, nucleoplasmic and chromatin fractions can be easily prepared from a pellet of cultured cells. In detail, 2 × 10^7^ ESCs were harvested by centrifugation, washed in PBS, and directly resuspended in 300 μL of Fractionation buffer (PARIS, #AM1921) supplemented with anti-protease, and the cells were incubated for 5 min on ice. Nuclei were collected in the pellet (P1) by low-speed centrifugation (5 min, 1500 rpm, 4 °C). The supernatant (S1: Cytoplasmic fraction) was further clarified by high-speed centrifugation (10 min, 14,000 rpm, 4 °C) to remove cell debris and insoluble aggregates. Nuclei (P1) were then lysed in 150 μL of Glycerol buffer (20 mM Tris-HCl, 75 mM NaCl, 0.5 mM EDTA, 0.85 mM DTT, 0.125 mM Phenylmethylsulfonyl fluoride, 50% Glycerol, pH 8.0) and 150 μL of Nuclei Resuspension buffer (10 mM HEPES, 1 mM DTT, 7.5 mM MgCl_2_, 0.2 mM EDTA, 0.3 M NaCl, 1 M Urea, 1% NP-40, pH 7.5). The supernatant (S2: Nucleoplasmic fraction) was further collected by high-speed centrifugation (5 min, 14,000 rpm, 4 °C). The insoluble chromatin pellet (P2) was resuspended in 50 μL of F-buffer and sonicated for three pulses (30 s “ON”, 30 s “OFF”, High). The supernatant (S3: Chromatin fraction) was further clarified by high-speed centrifugation (10 min, 14,000 rpm, 4 °C). The separated subcellular fractions were used for further assays and analysis.

### 4.6. Protein Extraction and Western Blotting

For total cell extracts, cells were resuspended in cold F-buffer (10 mM Tris-HCl pH 7.0, 50 mM NaCl, 30 mM Na-pyrophosphate, 50 mM NaF, 1% Triton X-100, anti-proteases), sonicated for three pulses (30 s “ON”, 30 s “OFF”, High), and then stored on ice for 10 min. Cell extract was then centrifuged for 10 min at 13,000 rpm, and the pellet was discarded. Extracts were quantified using BCA assay (Pierce, Waltham, MA, USA). Equivalent amounts of protein extract were separated by SDS-polyacrylamide gels (10% TGX Stain-Free Protein Gels, Bio-Rad) at different percentages, transferred to nitrocellulose membranes (iBlot™ 2 Transfer Stacks, Invitrogen, Waltham, MA, USA). Membranes were blocked with 5% milk in TBS and incubated with specific primary antibody overnight at 4 °C. Membranes were rinsed and incubated with HRP-conjugated secondary antibodies for 1 h at room temperature, followed by chemiluminescent detection using Amersham ECL Western Blotting Detection Reagent (GE Healthcare, Chicago, IL, USA) and Luminata™ Forte Western HRP Substrate (Millipore, Burlington, MA, USA). Primary antibodies used were: rabbit monoclonal IgG Biotin (5597s; Cell signaling, 1:1000, Danvers, MA, USA), mouse monoclonal IgG Vinculin (SAB4200080; Sigma, 1:5000, St. Louis, MI, USA), rabbit polyclonal IgG Histone H3 (ab1791; Abcam, 1:2000, Cambridge, UK), rabbit polyclonal IgG Avi-tag (A00674; GenScript, 1:1000, Piscataway, NJ, USA), mouse monoclonal IgG V5-tag (R961-25; Invitrogen, 1:1000, Waltham, MA, USA) and rabbit polyclonal IgG Smad7 (PA1-41506; Invitrogen, 1:1000, Waltham, MA, USA).

### 4.7. Immunofluorescence

Cells were prepared for immunofluorescence by fixation in 4% paraformaldehyde (Sigma, St. Louis, MI, USA) for 10 min and subsequent permeabilization for 15 min with 0.1% Triton X-100 in PBS at room temperature. Cells were then blocked for non-specific binding with 2% BSA in PBS for 2 h at room temperature. Primary antibodies were diluted in 0.1% BSA and incubated with the samples overnight at 4 °C. Samples were rinsed with PBS and incubated with the appropriate fluorescent dye-labeled secondary antibodies (Alexa Fluor; Invitrogen, 1:1000, Waltham, MA, USA) for 1 h at room temperature protected from light. Primary antibodies used were mouse monoclonal IgG Smad7 (sc-101152; Santa Cruz, 1:100, Dallas, TX, USA), and goat IgG Biotin (559286; BD Pharmingen, 1:100, Franklin Lakes, NJ, USA). Nuclei were visualized with DAPI (1 μg mL^−1^). The fluorescence intensity was calculated by using ImageJ software.

### 4.8. AP Staining

Cells were plated at a density of 5 × 10^3^/cm^2^ and after 2 days were fixed with 4% paraformaldehyde for 2 min and then stained with alkaline phosphatase (AP) solution (Vector^®^ Red alkaline phosphatase substrate kit; catalog No. SK-5100, Burlingame, CA, USA). The AP activity was calculated by using ImageJ software.

### 4.9. RNA Extraction and RT-qPCR

RNA samples were extracted directly from cultured cells using Trizol reagent (Invitrogen, Waltham, MA, USA) followed by isopropanol precipitation, and sample quality control was performed with Agilent Bioanalyzer 2100. All of the samples had an RNA integrity number ranging from 9.9 to 10. Briefly, mRNA quantitation was performed by RT-qPCR using the Superscript III platinum One-step RT-qPCR System kit (Invitrogen, Waltham, MA, USA) and normalized on *Actinb* mRNA. Oligonucleotide sequences are indicated in Table S2.

### 4.10. RNA-seq Library Preparation

For RNA-seq library preparation, 1 μg of total RNA was depleted of ribosomal RNA using the RiboMinus Eukaryote System v2 kit (Invitrogen, Waltham, MA, USA), following manufacturer instructions. Ribo-RNA was resuspended in 17 μL of EFP buffer (Illumina, San Diego, CA, USA), heated to 94 °C for 8 min, and used as input for the first-strand synthesis, using the TruSeq RNA Sample Prep kit, following manufacturer instructions. Poly(A) RNA-seq library was performed by using the TruSeq RNA Sample Prep kit, following the manufacturer’s instructions.

### 4.11. RNA-seq Analysis

Sequencing was performed on the Illumina NextSeq 500 platform. After quality controls with FastQC (https://www.bioinformatics.babraham.ac.uk/projects/fastqc/ (accessed on 17 December 2021)), raw reads were aligned to the GRCm38 mouse genome reference (mm10) using HiSat2 v2.2.0 [37] with options: -N 1 -L 20 -i S,1,0.5 -D 25 -R 5 --pen-noncansplice 20 --mp 1,0 --sp 3,0. Pre-built indexes based on the Ensembl transcript annotation (release 84) for guided alignment to transcriptome were retrieved from the HiSat2 website (https://cloud.biohpc.swmed.edu/index.php/s/grcm38_tran/download. (accessed on 17 December 2021)).

Gene expression levels were quantified with featureCounts v1.6.1 [38] (options: -t exon -g gene_name) using GENCODE comprehensive gene annotation (release M23—GRCm38.p6 https://www.gencodegenes.org/mouse/release_M23.html (accessed on 17 December 2021)). Multi-mapped reads were excluded from quantification. Gene expression counts were next analysed using the edgeR package [39]. After filtering lowly expressed genes (1 count per million (CPM) in less than 3 samples), normalization factors were calculated using the trimmed-mean of M-values (TMM) method (implemented in the calcNormFactors function) and CPM were obtained using normalized library sizes. Differential expression analysis was carried out by fitting a Generalized Linear Model (GLM) to all groups and performing Quasi-Likelihood F-test, while pairing each Smad7-shRNA with its respective scramble control (design matrix formula = “~treatment + batch”). Genes with False Discovery Rate (FDR) less than or equal to 0.05 were considered as differentially expressed. Hierarchical clustering of gene expression profiles was performed on differentially expressed genes only, using Euclidean distances and the complete linkage method. CPM values were scaled as Z-scores across samples before computing distances. Gene expression heatmaps were generated using the ComplexHeatmap R package.

### 4.12. Bio-ChIP-seq

Notably, 4 × 10^7^ ESCs expressed Bio-Smad7 protein were harvested, cross-linked with 1% formaldehyde for 10 min, quenched with 0.125 M glycine for 5 min, and then washed twice in cold 1× PBS. Cells were lysed in Isotonic Buffer (20 mM HEPES, 100 mM NaCl, 250 mM Sucrose, 5 mM MgCl_2_, 5 µM ZnCl_2_, pH 7.5) supplemented with 1% NP-40 and anti-protease. Cell lysates were centrifuged at 1000 rpm for 5 min at 4 °C to isolate the nuclei. Nuclei were suspended in 1 mL of 0.15% SDS Lysis Buffer (20 mM Tris-HCl, 150 mM NaCl, 2 mM EDTA, 0.15% SDS, 1% Triton X-100, pH 8.0) supplemented with anti-protease and subjected for sonication to shear chromatin fragments to an average size between 100 bp and 400 bp on the Pico Bioruptor^®^ (Diagenode, Denville, NJ, USA) (2 runs of 10 cycles (30 s “ON”, 30 s “OFF”) at high-power setting. Fragmented chromatin was centrifuged at 13,000 rpm for 15 min at 4 °C.

An amount of 50 µL MyOne^TM^ Streptavidin T1 Dynabeads (Thermo-Fisher Scientific, Waltham, MA, USA) was saturated with PBS/1% BSA at RT for 1 h and then incubated with the sample at 4°C overnight on a rotator. After incubation, Dynabeads were washed twice with 1 mL of Washing Buffer I (2% SDS), once with 1 mL of Washing Buffer II (50 mM HEPES, 500 mM NaCl, 0.1% Sodium Deoxycholate, 1% Triton X-100, 1 mM EDTA, pH 7.5), once with 1 mL Washing Buffer III (10 mM Tris-HCl, 250 mM LiCl, 0.5% NP-40, 0.5% Sodium Deoxycholate, 1 mM EDTA, pH 8.0), and once with 1 mL of TE buffer (10 mM Tris-HCl, 1 mM EDTA, pH 7.5). All the washing steps were performed at RT for 8 min on a rotator. The chromatin was eluted in 250 µL SDS Elution Buffer (1% SDS, 50 mM Tris-HCl, 10 mM EDTA, pH 8.0) followed by reverse cross-linking at 65 °C overnight. After decrosslinking, the ChIP DNA was purified using QIAQuick PCR Purification Kit (Qiagen, Hilden, Germany) according to the manufacturer’s instructions. For the reference sample, BirA-ESCs without tagged protein were used.

### 4.13. Bio-ChIP-seq Library Preparation

The purified ChIP DNA was processed for library generation using the NEBNext ChIP-seq Library Prep Master Mix (NEB, Ipswich, MA, USA) following the manufacturer’s protocol. Libraries were pooled and sequenced on an Illumina Next500 Platforms using the 75bp high output sequencing kit (Illumina, San Diego, CA, USA).

### 4.14. Bio-ChIP-seq Data Analysis

Following quality controls (performed with FastQC v0.11.2), sequencing reads were processed with Trim Galore! v0.5.0 (https://www.bioinformatics.babraham.ac.uk/projects/trim_galore (accessed on 17 December 2021)) to perform quality and adapter trimming (parameters: --stringency 3 –q 20). Trimmed reads were next analysed with the ENCODE Transcription Factors and Histone Modifications ChIP-seq pipeline 2 (v1.6.1, available online: https://github.com/ENCODE-DCC/chip-seq-pipeline2 (accessed on 17 December 2021)), using default software and parameter settings for the ‘Transcription Factors’ processing mode. Briefly, reads were aligned to the mouse reference genome (UCSC mm10) using Bowtie2 [40]. Duplicated, multi-mapping and poor-quality alignments were discarded, and peak calling was performed using MACS2 [41], using the input DNA as control library. Signal tracks were generated as fold enrichment over control for both individual and pooled replicates using MACS2. To determine a consensus set of Smad7 putative genomic binding sites conserved across biological replicates, the Irreproducible Discovery Rate (IDR) procedure was carried out, as implemented in the ENCODE pipeline [42]. Starting from overlapped peak calls in individual libraries (with SPP [43] peak caller at FDR < 0.01 and using the mock bioChIP-seq as control), the conservative set (i.e., from the comparison of true replicates) of IDR thresholded (IDR < 0.05) peaks was retained as the final set of Smad7 binding sites. Genomic annotation of Smad7 peaks was carried out using the HOMER [44] suite (annotatePeaks.pl utility). Motif discovery and motif enrichment analyses were preformed using the HOMER [44] suite (findMotifsGenome.pl). Association of Smad7 peaks to regulatory elements was performed using the annotated list of Candidate *cis*-Regulatory Elements (cCRE) for mESC cell line E14 available from the SCREEN (ENCODE Project Consortium, 2010) database. Heatmaps of ChIP-seq signals over regulatory regions were generated using the deepTools [45] computeMatrix and plotHeatmap utilities. Motif discovery analysis was performed using the HOMER suite (findMotifsGenome.pl utility). ChIP-seq datasets for comparative analysis in mESC E14 were retrieved from ENCODE for histone marks/chromatin accessibility, and from GEO for transcription factors (accession code GSE11431, GSE125116). Correlation heatmaps were generated on a merged set of binding sites (defined by the ENCODE pipeline or by MACS2 [41] at *q*-value < 0.05 for the GEO-retrieved datasets) using the deepTools multiBamSummary and plotCorrelation utilities, using Spearman correlation and input-normalized ChIP-seq signals for sample clustering. To reconstruct the regulatory network of pluripotency-associated transcription factors (TFs), the directed regulatory interactions (i.e., network edges) between each TF and its target genes were inferred by (i) filtering ChIP-seq binding peaks for their occurrence in promoter or enhancer-like signatures (as defined by SCREEN cCREs) and (ii) associating the peaks to target genes with GREAT [46].

### 4.15. Bio-ChIP Assay

For ChIP-qPCR analysis, 1 × 10^7^ ESCs transduced with BirA and linearized Smad7 constructs were used. The immunoprecipitated DNA was isolated using the protocol described for Bio-ChIP and was analyzed by quantitative PCR (qPCR) using the SYBR GreenER kit (Invitrogen, Waltham, MA, USA). For input samples, 250 µL of SDS Elution Buffer was added into 20 µL of the sheared chromatin. The samples were incubated at 65 °C overnight to reverse cross-linking. DNA fragments were purified with the QIAquick PCR Purification Kit and eluted with 40 µL of H_2_O. Quantitative PCR was performed with approximately 2% of ChIP sample. The amount of each amplification product was determined relative to a standard curve, and fold enrichment was calculated by comparison of amplified product from the bioChIP sample and ChIP samples from BirA containing ESCs. The amount of immunoprecipitated DNA was calculated relative to input. Primer pairs for quantitative ChIP-PCR were designed using ±150 bp genomic sequence information specific to the predicted target loci to generate 100 bp to 200 bp amplified products. Primers targeting mouse promoter sequences were used for qPCR analysis. Primer sequences are listed in Table S2.

### 4.16. Published Datasets

Datasets were obtained from Gene Expression Omnibus for ESCs histone modifications (GSE12241, GSE11172, and GSE31039) and transcription factors (GSE11431, GSE125116).

## Data Availability

Datasets are from Gene Expression Omnibus for ESCs histone modifications (GSE12241, GSE11172, and GSE31039) and transcription factors (GSE11431, GSE125116). The dataset that supports the findings of this study are in GSE185905.

## Supplementary Materials

The following are available online at https://www.mdpi.com/article/10.3390/ijms222413598/s1.
