# Supplementary material for: Genome-Wide Analysis of Smad7-Mediated Transcription in Mouse Embryonic Stem Cells
Source: Int J Mol Sci. 2021 Dec 18;22(24):13598. doi: 10.3390/ijms222413598 (PMC8708723; doi:10.3390/ijms222413598)
Supplement: Supplementary file 1 [file ijms-22-13598-s001.zip › ijms-1487796-supplementary.pdf]

Guohua Meng et al.

Genome-wide Analysis of Smad7-Mediated Transcription in Mouse Embryonic Stem Cells

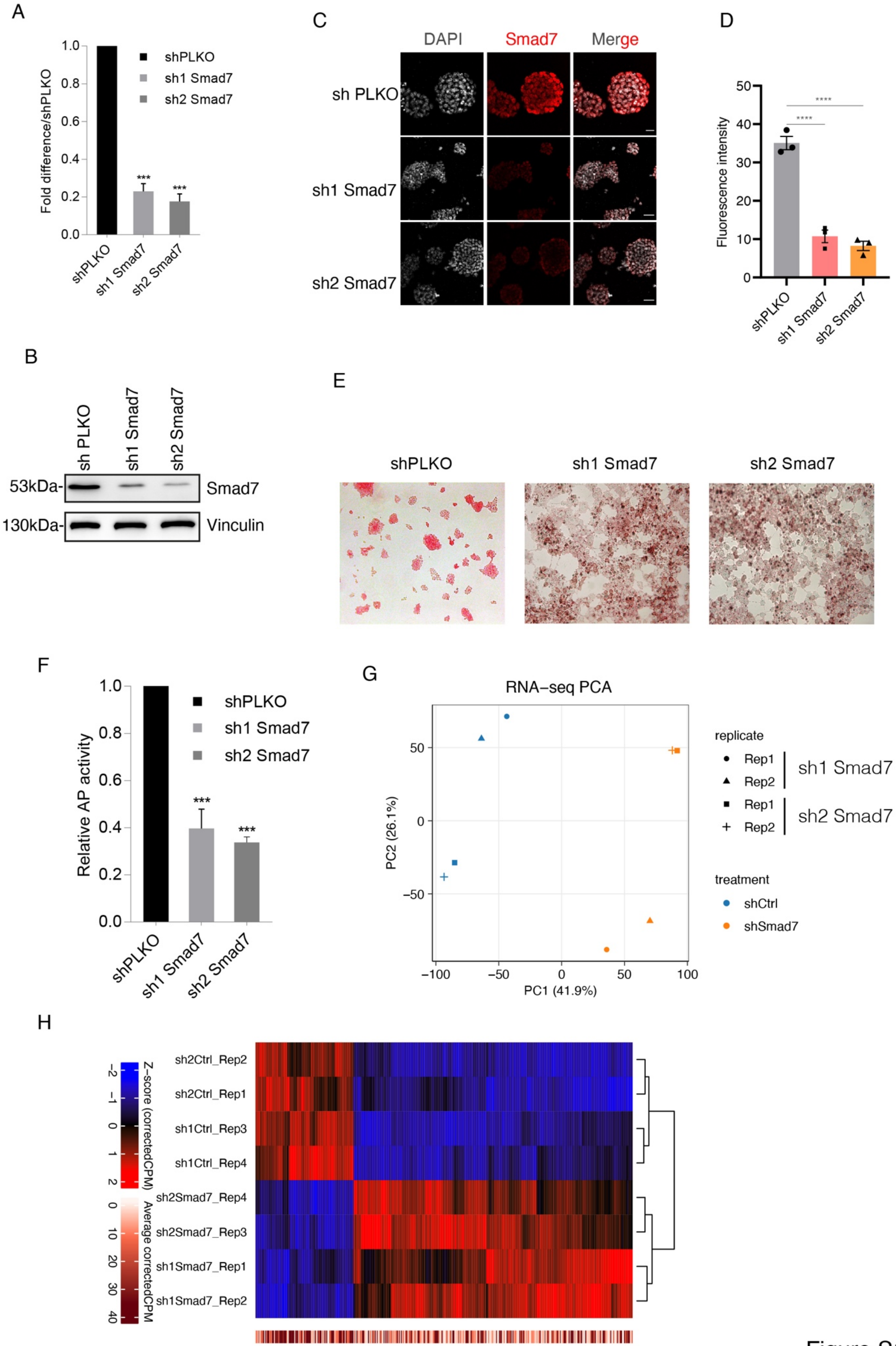
A
Fold difference/shPLKO
1.0
0.8
0.6
0.4
0.2
0.0
shPLKO
sh1 Smad7
sh2 Smad7
***
***
B
sh PLKO
sh1 Smad7
sh2 Smad7
53kDa-
130kDa-
Smad7
Vinculin
C
DAPI
Smad7
Merge
sh PLKO
sh1 Smad7
sh2 Smad7
D
Fluorescence intensity
50
40
30
20
10
0
****
****
shPLKO
sh1 Smad7
sh2 Smad7
E
shPLKO
sh1 Smad7
sh2 Smad7
F
Relative AP activity
1.0
0.8
0.6
0.4
0.2
0.0
shPLKO
sh1 Smad7
sh2 Smad7
***
***
G
RNA−seq PCA
PC2 (26.1%)
PC1 (41.9%)
50
0
−50
−100
−50
0
50
100
replicate
Rep1
Rep2
sh1 Smad7
Rep1
Rep2
sh2 Smad7
treatment
shCtrl
shSmad7
H
Z-score (correctedCPM)
−2
−1
0
1
2
Average correctedCPM
0
10
20
30
40
sh2Ctrl_Rep2
sh2Ctrl_Rep1
sh1Ctrl_Rep3
sh1Ctrl_Rep4
sh2Smad7_Rep4
sh2Smad7_Rep3
sh1Smad7_Rep1
sh1Smad7_Rep2

Figure S1

Supplementary Figure 1. RNA-seq analysis showing the silencing of Smad7 regulates the expression of the transcripts in mESCs.

A. RT-qPCR analysis of Smad7 expression in ESCs. Data are shown as mean ± SEM; n = 3. The results are shown as fold difference normalized to shPLKO. ***P <0.001.
B. Western blot showing the knockdown of Smad7 after transfecting shRNA of Smad7. Cells were harvested 48 hours after the transfection.
C. Representative confocal fluorescence images of Smad7 (red) in the ESCs transfected with 2 different Smad7 shRNAs compared with the shPLKO control. The nuclei were counter-stained with DAPI (grey). Scale bar = 50 μm.
D. Quantification of fluorescence intensity of Smad7 expression in the ESCs transfected with 2 different Smad7 shRNAs compared with the shPLKO control. n = 3, ****, P < 0.0001.
E. AP staining of ESCs wild type and two independent clones expressing shSmad7.
F. Quantification of the AP positive colonies. Data are shown as mean ± SEM; n = 3. The results are shown as fold difference normalized to shPLKO. **P <0.01.
G. Principal component analysis (PCA) shows clustering of RNA-seq samples by treatment and replicates. While PC1, explaining 41.9 % of the total variance, separates treated samples from control samples, PC2, explaining 26.1 % of the total variance, differentiates the replicates.
H. RNA-seq heatmap displaying the differentially expressed genes when comparing the knockdown of Smad7 (shSmad7) with control (shPLKO). Four replicates for each were used in this experiment. The significance threshold is log2 fold change > 0.5 and FDR < 0.05. Z-scores represented relative expressions to generate the heatmap.

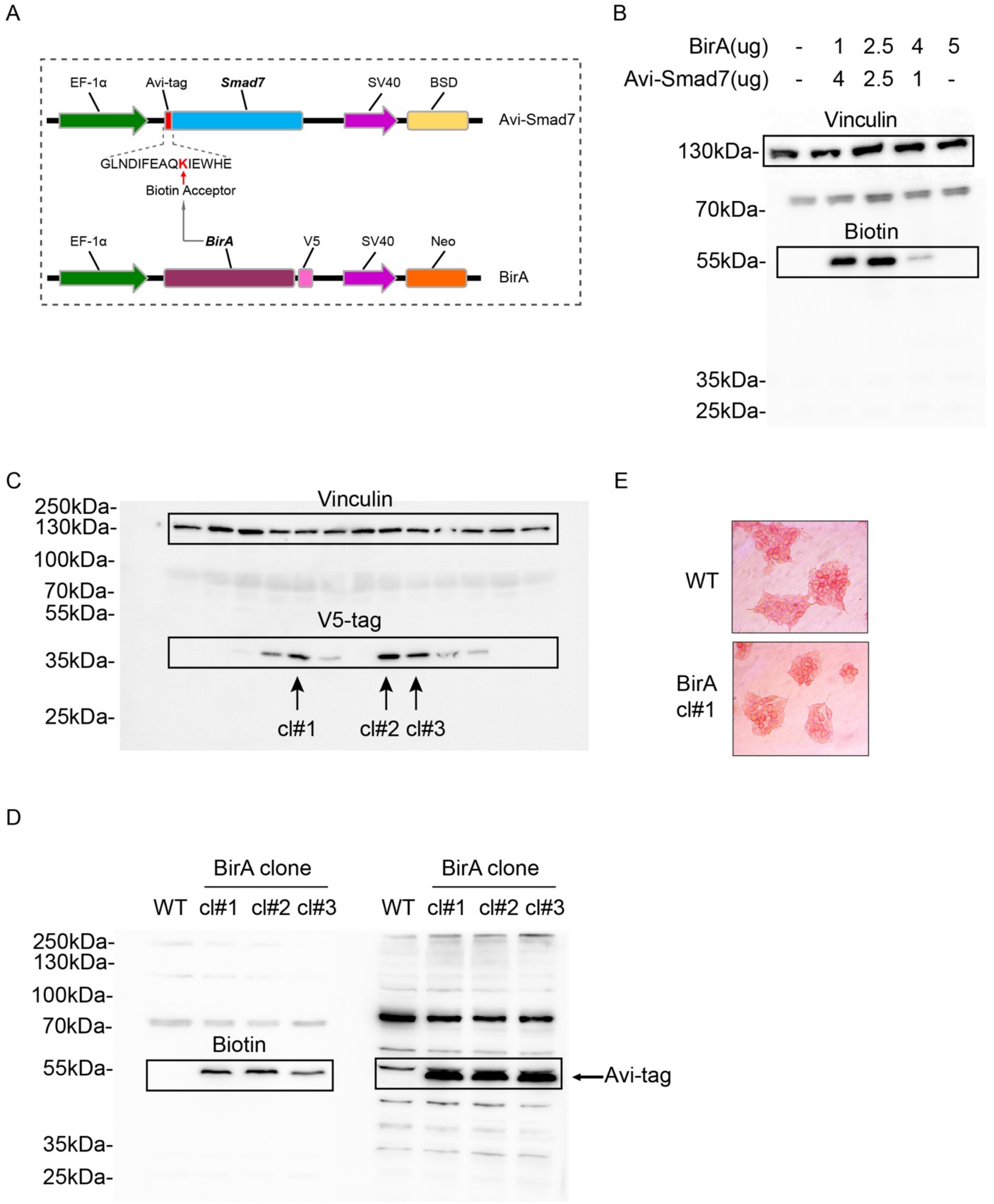
A
EF-1α
Avi-tag
Smad7
SV40
BSD
Avi-Smad7
GLNDIFEAQKIEWHE
Biotin Acceptor
EF-1α
BirA
V5
SV40
Neo
BirA
B
BirA(ug) - 1 2.5 4 5
Avi-Smad7(ug) - 4 2.5 1 -
Vinculin
130kDa-
70kDa-
Biotin
55kDa-
35kDa-
25kDa-
C
250kDa-
130kDa-
100kDa-
70kDa-
55kDa-
35kDa-
25kDa-
Vinculin
V5-tag
cl#1
cl#2 cl#3
E
WT
BirA
cl#1
D
BirA clone
BirA clone
WT cl#1 cl#2 cl#3
WT cl#1 cl#2 cl#3
250kDa-
130kDa-
100kDa-
70kDa-
55kDa-
35kDa-
25kDa-
Biotin
Avi-tag

Figure S2

Supplementary Figure 2. Characterization and functional validation of BirA-ES stable clone.

A. Schematic of the two components of the *in vivo* biotinylation system: an Avi-Smad7 vector and a biotin ligase BirA.

B. Western blot using an anti-Biotin antibody comparing the expression of biotinylated Smad7 with different combinations of the transfected expression vector. Five ug vector was used for each transfection.

C. Western blot using an anti-V5-tag antibody screened the expression of BirA in the stable clones. Vinculin was used as a loading control.

D. Western blot showing the overexpression of Smad7 after transfecting Avi-Smad7 vector in three different BirA-ES clones. Cells were harvested 30 hours after the transfection. Biotin and Avi-tag antibody were used to determine the transfection efficiency. E.

AP staining of WT and BirA ESCs.

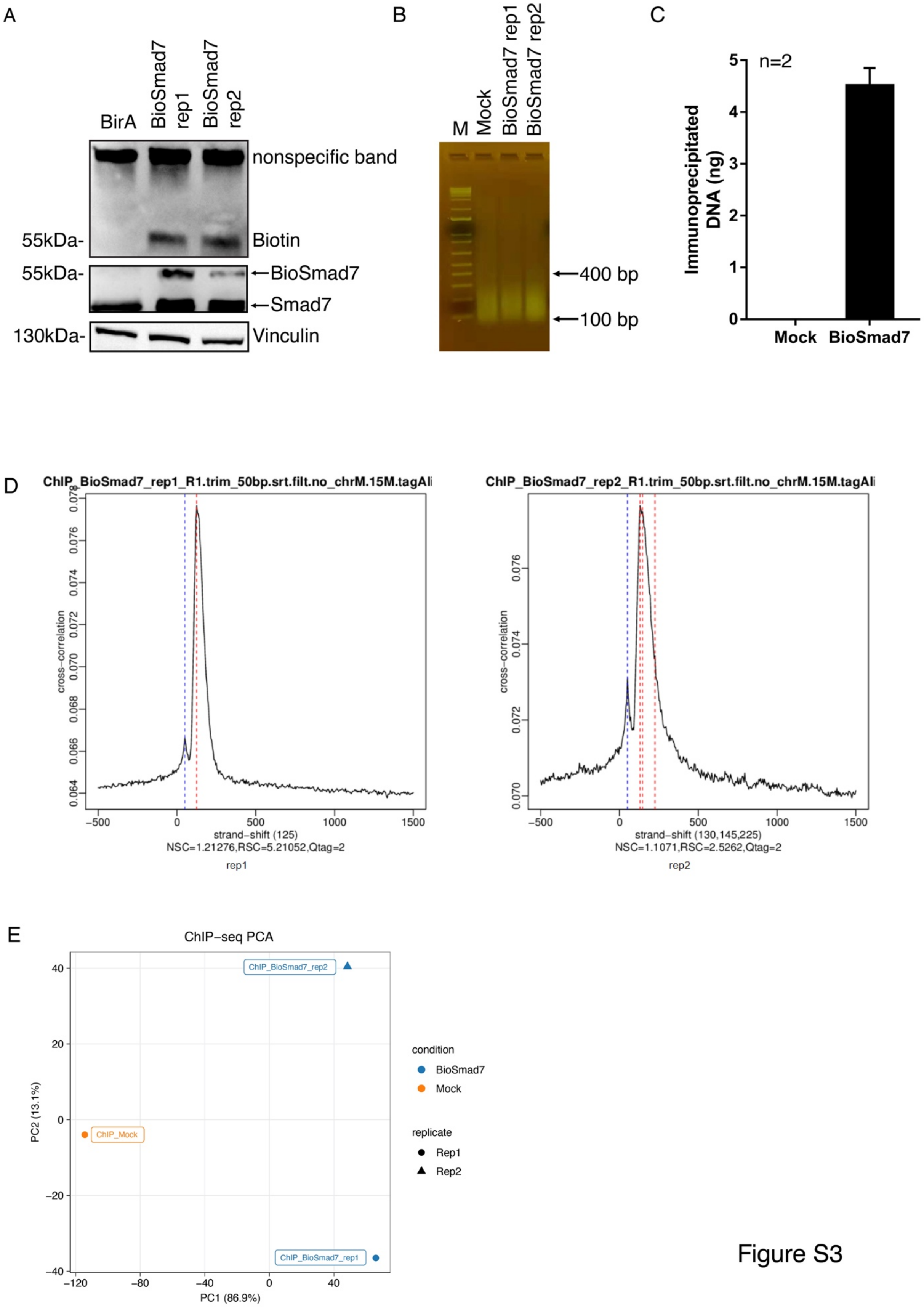

A
BirA
BioSmad7 rep1
BioSmad7 rep2
nonspecific band
55kDa-
Biotin
55kDa-
←BioSmad7
←Smad7
130kDa-
Vinculin
B
M
Mock
BioSmad7 rep1
BioSmad7 rep2
←400 bp
←100 bp
C
n=2
Immunoprecipitated DNA (ng)
0
1
2
3
4
5
Mock
BioSmad7
D
ChIP_BioSmad7_rep1_R1.trim_50bp.srt.filt.no_chrM.15M.tagAli
cross−correlation
strand−shift (125)
NSC=1.21276,RSC=5.21052,Qtag=2
rep1
ChIP_BioSmad7_rep2_R1.trim_50bp.srt.filt.no_chrM.15M.tagAli
cross−correlation
strand−shift (130,145,225)
NSC=1.1071,RSC=2.5262,Qtag=2
rep2
E
ChIP−seq PCA
ChIP_BioSmad7_rep2
ChIP_Mock
ChIP_BioSmad7_rep1
PC1 (86.9%)
PC2 (13.1%)
condition
BioSmad7
Mock
replicate
Rep1
Rep2

Figure S3

Supplementary Figure 3. An overview of the experimental procedures for BioChIP of Smad7 in mESCs.

A. Western blot shows the in vivo biotinylation of Smad7. Biotin antibody was used to determine the expression of biotinylated Smad7. The native antibody was used against both endogenous and biotinylated proteins. The subendogenous level of the biotinylated Smad7 (BioSmad7) and the expression level of the endogenous Smad7 (EndoSmad7) are indicated. Vinculin was used as a loading control. Two replicates were used in this experiment.
B. Agarose gel electrophoresis shows the sonicated chromatin extracts with reversed crosslinking. 1 ml of nuclear extract was sonicated for 20 minutes using a Pico bioruptor with the "HIGH" setting, cycled 30 sec on and 30 sec off. The size of the sonicated chromatin ranges from 100 bp to 400 bp.
C. The bar graph shows the immunoprecipitated DNA after the BioChIP assay. Mock (BirA cells) was used as a negative control. Two replicates were used here.
D. Two replicates of the BioChIP libraries were assessed on a bioanalyzer.
E. Principal component analysis (PCA) analysis showing two replicates of BioChIP-seq of Smad7 and the Mock. Note that the first PCA component (PC1) explained > 86.9 % of the variation, and the PC1 values for the two replicates were similar.

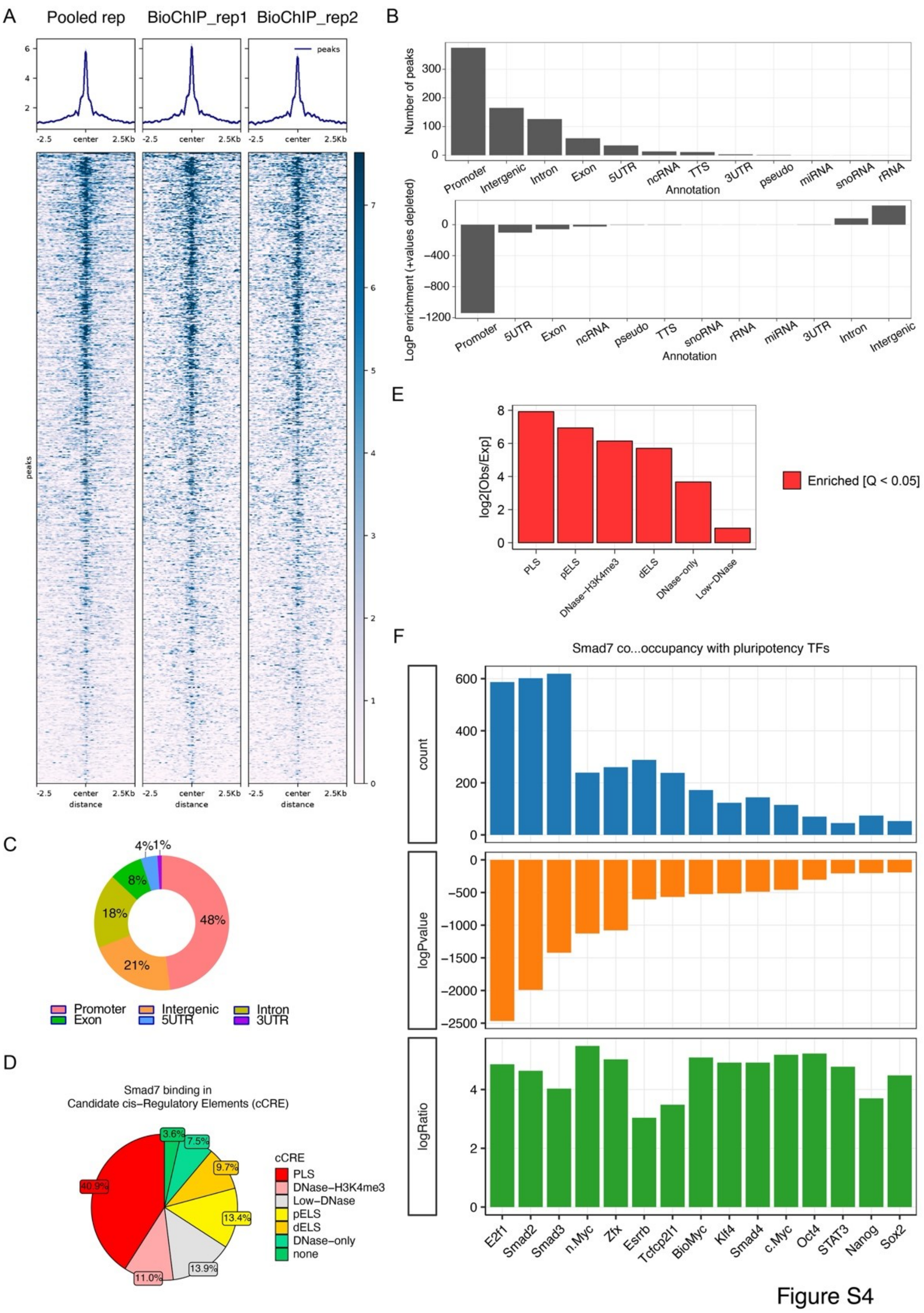

A
Pooled rep
BioChIP_rep1
BioChIP_rep2
peaks
center
distance
B
Number of peaks
Promoter
Intergenic
Intron
Exon
5UTR
ncRNA
TTS
3UTR
pseudo
miRNA
snoRNA
rRNA
Annotation
LogP enrichment (+values depleted)
C
48%
21%
18%
8%
4%
1%
Promoter
Exon
Intergenic
5UTR
Intron
3UTR
D
Smad7 binding in
Candidate cis−Regulatory Elements (cCRE)
cCRE
PLS
DNase−H3K4me3
Low−DNase
pELS
dELS
DNase−only
none
E
log2[Obs/Exp]
Enriched [Q < 0.05]
F
Smad7 co...occupancy with pluripotency TFs
count
logPvalue
logRatio
E2f1
Smad2
Smad3
n.Myc
Zfx
Esrrb
Tcfcp2l1
BioMyc
Klf4
Smad4
c.Myc
Oct4
STAT3
Nanog
Sox2

Figure S4

Supplementary Figure 4. Statistics on the Smad7 ChIP-seq analysis.

A. Heatmaps show the colocalization of Smad7 ChIP-seq peaks in pooled replicate, replicate 1 and replicate 2. Occupancy signals within ± 2.5 Kb of the center of Smad7-binding sites are shown.
B. Bar graphs present the distribution of Smad7 binding peaks across the mouse genome. Promoters: regions within ± 2.5 kb from the TSSs; Intergenic: regions except for promoters, exons, and introns.
C. Distribution of BioSmad7 ChIP-seq peaks across the mouse genomic regions.
D. A pie chart is presenting the distribution of Smad7 binding sites in candidate *cis*-regulatory elements (cCRE). This data is corresponding to Figure 2D.
E. Barplot showing significant enrichment (Q-value < 0.05) of Smad7 peaks over cCREs.
F. Bar graphs show Smad7 (current study) co-occupancy with other TFs (accession code GSE11431, GSE125116) in ESC.

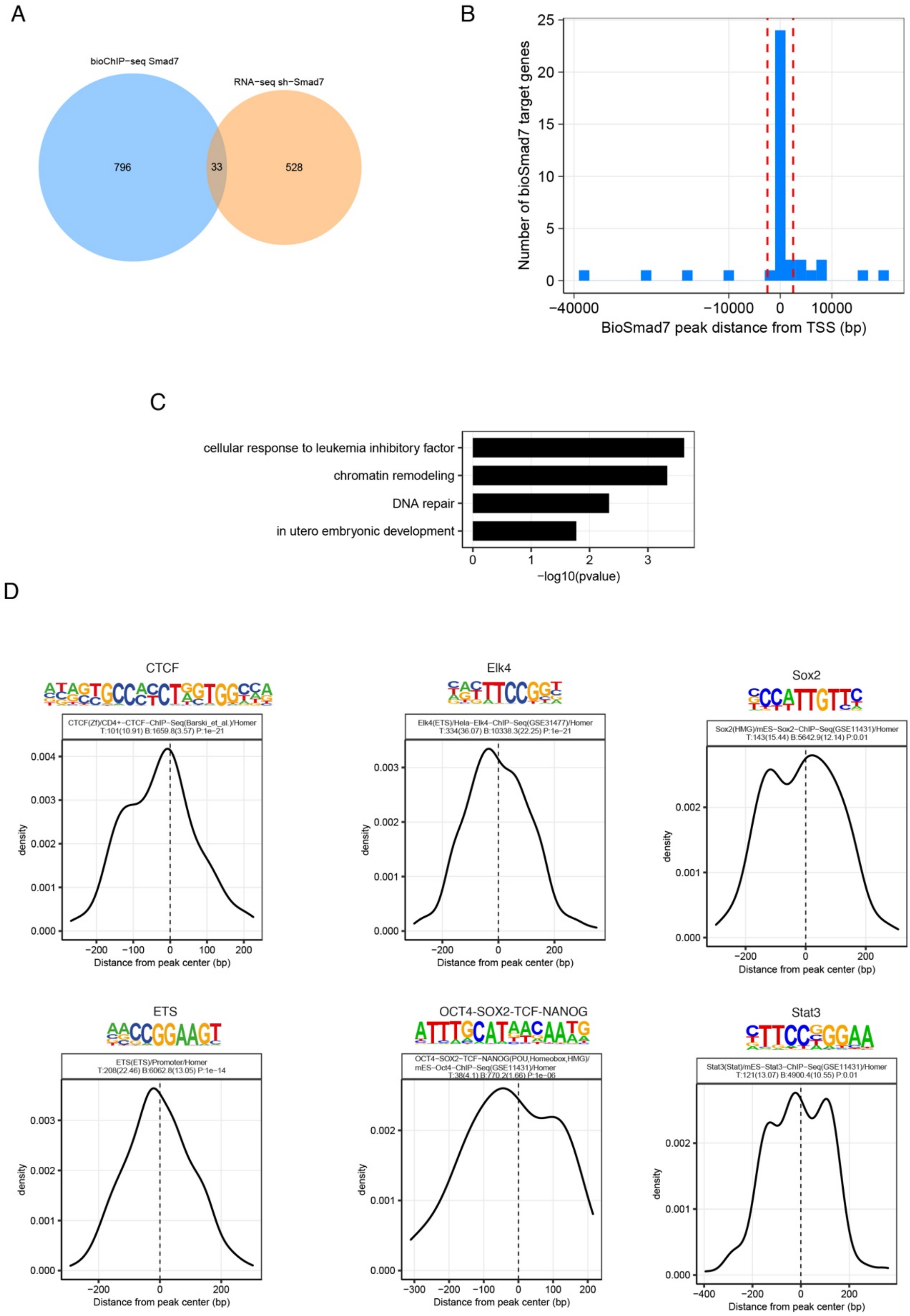
A
bioChIP−seq Smad7
RNA−seq sh−Smad7
796
33
528
B
Number of bioSmad7 target genes
BioSmad7 peak distance from TSS (bp)
C
cellular response to leukemia inhibitory factor
chromatin remodeling
DNA repair
in utero embryonic development
−log10(pvalue)
D
CTCF
CTCF(Zf)/CD4+−CTCF−ChIP−Seq(Barski_et_al.)/Homer
T:101(10.91) B:1659.8(3.57) P:1e−21
Elk4
Elk4(ETS)/Hela−Elk4−ChIP−Seq(GSE31477)/Homer
T:334(36.07) B:10338.3(22.25) P:1e−21
Sox2
Sox2(HMG)/mES−Sox2−ChIP−Seq(GSE11431)/Homer
T:143(15.44) B:5642.9(12.14) P:0.01
ETS
ETS(ETS)/Promoter/Homer
T:208(22.46) B:6062.8(13.05) P:1e−14
OCT4-SOX2-TCF-NANOG
OCT4−SOX2−TCF−NANOG(POU,Homeobox,HMG)/
mES−Oct4−ChIP−Seq(GSE11431)/Homer
T:38(4.1) B:770.2(1.66) P:1e−06
Stat3
Stat3(Stat)/mES−Stat3−ChIP−Seq(GSE11431)/Homer
T:121(13.07) B:4900.4(10.55) P:0.01
density
Distance from peak center (bp)

Figure S5

Supplementary 5

A. Venn diagram indicated the overlapped genes between downregulated genes (FDR < 0.05) after Smad7 knockdown and Smad7 ChIP-seq data.
B. Bar graph representing the enriched GO terms (P-value < 0.05) of biological processes in the overlapped genes between downregulated genes after Smad7 knockdown and Smad7 ChIP-seq data.
C. Histogram showing the distribution of Smad7 ChIP-seq binding sites relative to TSSs of regulated genes after Smad7 knockdown. Red dash lines indicate the promoters (regions within ± 2.5 kb from the TSSs).
D. The consensus sequences and spatial distributions of motifs of other transcription factors that are the most enriched in Smad7 binding sites.

SUPPLEMENTARY DATA

File Name: Supplementary Table S1

Description: Statistics for Bowtie mapping and peak calling in Smad7 ChIP-seq analysis.

File Name: Supplementary Table S2

Description: Oligonucleotide sequences used as primers with indication of the corresponding gene.

File Name: Supplementary Data 1

Description: Significantly differentially expressed gene list after the silencing of Smad7.

File Name: Supplementary Data 2

Description: Smad7 binding target list after the peak-calling by using two different methods.

File Name: Supplementary Data 3

Description: Overlapped genes between Smad7 binding targets and RNA-seq data on the silencing of Smad7.

File Name: Supplementary Data 4
Description: Gene list of Smad7 direct targets and indirect targets.
